# Gut mucosal microbiome across stages of colorectal carcinogenesis

**DOI:** 10.1038/ncomms9727

**Published:** 2015-10-30

**Authors:** Geicho Nakatsu, Xiangchun Li, Haokui Zhou, Jianqiu Sheng, Sunny Hei Wong, William Ka Kai Wu, Siew Chien Ng, Ho Tsoi, Yujuan Dong, Ning Zhang, Yuqi He, Qian Kang, Lei Cao, Kunning Wang, Jingwan Zhang, Qiaoyi Liang, Jun Yu, Joseph J. Y. Sung

**Affiliations:** 1 Department of Medicine and Therapeutics, Institute of Digestive Disease, State Key Laboratory of Digestive Disease, Li Ka Shing Institute of Health Sciences, The Chinese University of Hong Kong, 30-32 Ngan Shing Street, Shatin, Hong Kong SAR, China; 2 CUHK Shenzhen Research Institute, 2 Yuexing Road, Nanshan District, Shenzhen 518057, China; 3 Department of Microbiology, The Chinese University of Hong Kong, 30-32 Ngan Shing Street, Shatin, Hong Kong SAR, China; 4 Department of Gastroenterology, Beijing Military General Hospital, 28 Fuxing Road, Haidian, Beijing 100853, China; 5 Department of Anaesthesia and Intensive Care, The Chinese University of Hong Kong, 30-32 Ngan Shing Street, Shatin, Hong Kong SAR, China; 6 Department of Gastroenterology, The First Affiliated Hospital of Sun Yat-sen University, 58 Zhongshan Second Road, Yuexiu, Guangzhou 510080, China

## Abstract

Gut microbial dysbiosis contributes to the development of colorectal cancer (CRC). Here we catalogue the microbial communities in human gut mucosae at different stages of colorectal tumorigenesis. We analyse the gut mucosal microbiome of 47 paired samples of adenoma and adenoma-adjacent mucosae, 52 paired samples of carcinoma and carcinoma-adjacent mucosae and 61 healthy controls. Probabilistic partitioning of relative abundance profiles reveals that a metacommunity predominated by members of the oral microbiome is primarily associated with CRC. Analysis of paired samples shows differences in community configurations between lesions and the adjacent mucosae. Correlations of bacterial taxa indicate early signs of dysbiosis in adenoma, and co-exclusive relationships are subsequently more common in cancer. We validate these alterations in CRC-associated microbiome by comparison with two previously published data sets. Our results suggest that a taxonomically defined microbial consortium is implicated in the development of CRC.

## Abstract (web summary)

Changes in gut microbial communities contribute to the development of colorectal cancer. Here, the authors analyse the gut mucosal microbiome of patients and healthy subjects and identify distinct microbial consortia associated with different stages of colorectal cancer tumorigenesis.

## Graphical abstract

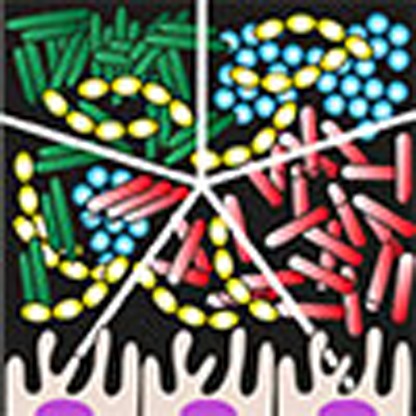

The human intestinal mucosa is a dynamic interface between host cells and a network of microbial ecosystems^1^. Sustained gut microbial dysbiosis is a potential risk factor for exacerbating colorectal lesions towards carcinogenesis^2^. Progression of colorectal neoplasia has been linked to alterations of tumour microenvironment and mucosal barrier function, which facilitate the interaction of microbial products with host pathways^3^. A variety of gut commensals and their metabolites, such as butyrate and hydrogen sulphide^4^,^5^, are known for triggering inflammatory cascades and oncogenic signalling, thereby promoting genetic and epigenetic alterations in the development of colorectal cancer (CRC)^3^. Given our lack of understanding on how microbiome profiles change during the transition from normal mucosae, adenomatous to malignant lesions, assigning certain members or a consortium of the gut microbes with potential causative roles in CRC remains a grand challenge. Although the enrichment of *Fusobacterium* species and their regulation of tumour microenvironment have been described^6^,^7^,^8^,^9^,^10^,^11^, increasing evidence suggests that colorectal lesions are home to various other members of the gut microbiota^12^,^13^,^14^. Thus, variations in the taxonomic footprints of microbial communities across major stages of CRC development need to be clarified.

Here we perform 16S ribosomal RNA (rRNA) gene sequencing on mucosal microbiome of normal colorectal mucosae, adenomatous polyps and adenocarcinomas. Our approach focuses on the identification of distinct taxonomic configurations, or metacommunities. To determine associations of metacommunities with disease status, we adopt an approach similar to that published by Ding and Schloss^15^. By further analyses of paired samples and microbial relationships, we demonstrate that mucosal microbial communities show distinct alterations across stages of colorectal carcinogenesis.

## Results

### Metacommunities associated with colorectal tumour statuses

**Figure 1:**
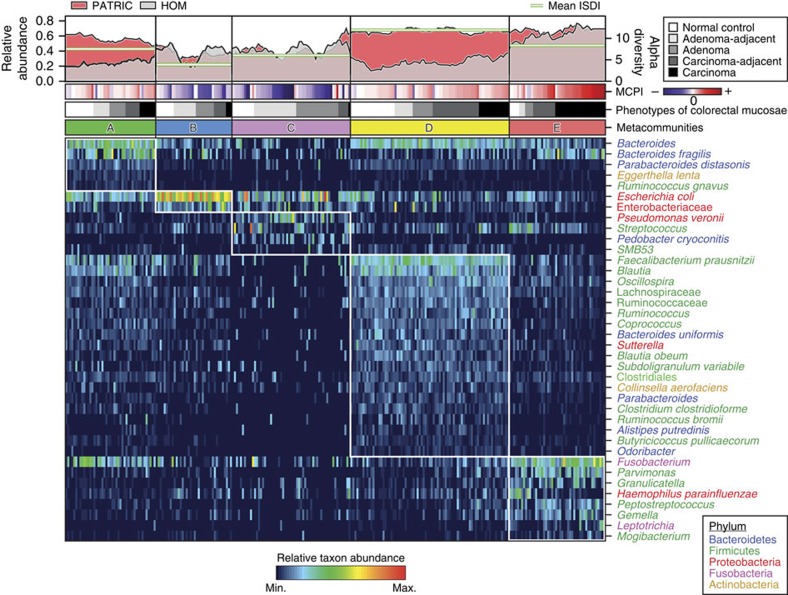
Characterization of 16S rRNA gene catalogue for mucosal microbial communities in colorectal carcinogenesis. Fitting microbiome data to DMM models defined five metacommunities. Reads that are considered as being potentially originated from oral strains or known pathogenic strains in the human gut were classified against the 16S rRNA gene collections from the Human Oral Microbiome (HOM; version 13) database and PATRIC bacterial pathogen database as defined by pseudo-bootstrapped (*n*=1,000) confidence scores of 100 at species-level taxa or deeper, using the naive Bayesian classifier. The panels of metacommunity markers are ranked in the descending order of linear discriminant analysis scores from top to bottom. Columns represent microbiome profiles (arcsine square root-transformed) of 269 mucosal biopsies from individuals with or without adenoma or adenocarcinomas. (MCPI<0 for changes characteristic of adenomas; MCPI>0 for changes characteristic of carcinomas).

To determine associations of microbiome profiles with mucosal phenotypes, we performed 16S rRNA gene sequencing on mucosal biopsy samples collected from subjects with normal colons (*n*=61), subjects with histology-proven adenoma (*n*=47), and subjects with invasive adenocarcinoma (*n*=52) at the Prince of Wales Hospital of the Chinese University of Hong Kong and the First Affiliated Hospital of the Sun Yat-Sen University (see Supplementary Table 1 for overview of patient demographics). We implemented the sequence curation pipeline optimized for analyses of amplicon libraries as described in mothur software package^16^. This approach for quality control has been shown to result in a low sequencing error rate (0.06% or less)^17^. Using the reference Greengenes taxonomies (version 13.8), post-quality control reads were assigned to bacterial phylotypes. Phylotypes with the deepest taxonomic annotations were fitted to Dirichlet multinomial mixture (DMM) models to partition microbial community profiles into a finite number of clusters, using the Laplace approximation as previously described (see Supplementary Fig. 1 for comparison of ordination results from DMM and partitioning around medoid (PAM)-based clustering)^15^,^18^. We identified five metacommunities designated in Fig. 1 as ‘A–E' and observed strong associations with phenotypes of colorectal mucosae (Fisher's exact test with Monte Carlo simulation; *q*=7.0 × 10^−5^; see also Supplementary Data 1 for associations of metacommunities with clinical features). Subsequently, we screened for taxa that distinguished the metacommunities using the LEfSe algorithms (Fig. 1; see Supplementary Data 2 for the summary of linear discriminant analysis scores), and performed receiver operating characteristic analyses to confirm that these markers confidently differentiated normal mucosae from lesions (Supplementary Fig. 2). The performance of metacommunity markers was comparable to that of the markers identified by Random Forests (see Supplementary Data 3 for the list of markers selected by tenfold cross-validations of the Random Forests algorithm)^19^. Furthermore, using metacommunity markers, we designed a two-way index, termed Microbial Community Polarization index (MCPI), to quantify the degree of mucosal dysbiosis associated with colorectal lesions (Fig. 1; see Supplementary Fig. 2a for the performance of the index).

Metacommunity A was represented by phylotypes of major bacterial phyla, including Bacteroidetes, Firmicutes, Proteobacteria and Fusobacteria. The representative members included *Bacteroides*, *Bacteroides fragilis*, *Fusobacterium*, *Escherichia coli*, *Faecalibacterium prausnitzii* and *Blautia* (Fig. 1). Metacommunity B was predominated by *E. coli* and had the least diverse community profile (Mann–Whitney *U*-test; mean *q*=1.5 × 10^−4^; Supplementary Fig. 1). Metacommunity C differed by high intra-cluster variability due to inconsistent appearances of taxa (Supplementary Fig. 1). Metacommunity D was overrepresented by members of the Firmicutes with *Bacteroides* being equally abundant. Of all the metacommunity compositions examined, metacommunity E was particularly interesting in that *Fusobacterium* as well as some other Firmicutes associated with periodontal diseases were enriched. Indeed, metacommunity E had significantly higher levels of oral and/or potentially pathogenic taxa sharing nearly identical sequences with the reference 16S rRNA genes from the Human Oral Microbiome (Mann–Whitney *U*-test; mean *q*=1.1 × 10^−3^; Supplementary Data 4) and the PATRIC bacterial pathogen databases (Mann–Whitney *U*-test; mean *q*=8.4 × 10^−3^; Supplementary Data 4). Metacommunities C and E were strongly associated with adenomas and carcinomas (Fisher's exact test; *q*<1.0 × 10^−5^), respectively. In total, 40% of adenomas were classified as metacommunity C whereas 48% of carcinomas were classified as metacommunity E. Metacommunities A and D together represented 59% of the normal controls.

**Figure 2:**
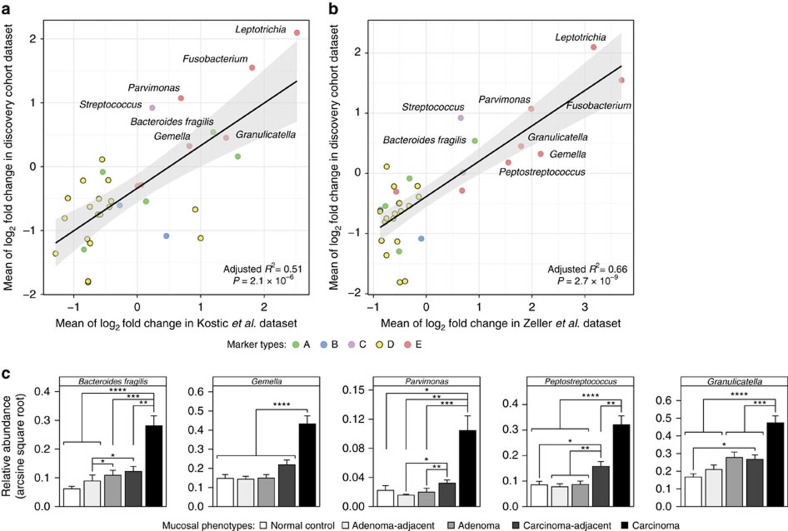
Validations of metacommunity markers in independent cohorts. (**a**,**b**) Fold-change analyses in paired carcinoma and carcinoma-adjacent samples in two additional cohorts demonstrated significant agreement with our discovery cohort: (**a**) Kostic *et al.*^7^ data set (*n*=74) and (**b**) Zeller *et al.*^20^ data set (*n*=48). Shown are adjusted *R*^2^ and *P* values for goodness of fit from multiple linear regression models. (**c**) Real-time PCR amplifications of the most abundant sequences of representative bacterial phylotypes showed consistent enrichments in an additional Chinese cohort consisting of 207 mucosal biopsies (normal control, *n*=25; adenoma, *n*=41; adenocarcinoma, *n*=50). Error bars represent s.e.m. *P* values from Mann–Whitney *U*-tests are adjusted by Benjamini-Hochberg (BH) step-up procedure; *q<0.05; **q<0.01; ***q<0.001; ****q<0.0001.

To validate the consistent enrichments of metacommunities in independent cohorts, we analysed the publicly available data sets of similar experimental design^7^,^20^. By training logistic regression models with LASSO penalization^21^ on relative abundance profiles in our discovery cohort that was previously subjected to DMM partitioning, we classified these independent samples into the metacommunities ‘A–E'. Fisher's exact tests showed that the enrichment of metacommunity E and depletion of metacommunity D in carcinomas are significant and consistent in both studies (Fisher's exact test; *q*<0.005 for both data sets; Supplementary Data 5). For metacommunity markers, we fitted multiple linear regression models to their fold changes in carcinoma relative to corresponding carcinoma-adjacent mucosa and demonstrated statistically significant agreements between our discovery cohort and the two studies (Fig. 2a,b). Furthermore, we performed real-time PCR amplification of the most abundant 16S rRNA marker gene sequences of representative bacterial phylotypes in an independent Chinese cohort comprising 116 individuals (normal colon, *n*=25; adenoma-affected, *n*=41; carcinoma-affected, *n*=50; see Supplementary Table 2 for overview of patient demographics) and confirmed the consistent enrichments of these markers (Fig. 2c).

### Paired analysis of mucosal metacommunities

**Figure 3:**
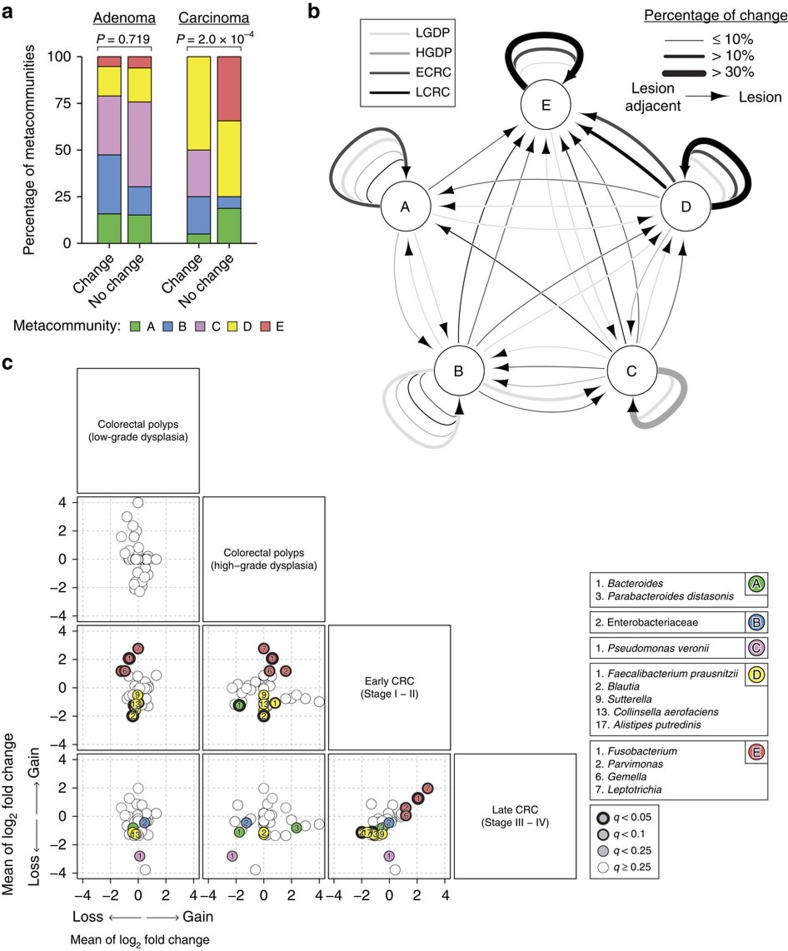
Community-wide alterations of microbiome profiles are important aspects of multistage colorectal tumour progression. (**a**) Discordance of taxonomic configurations between lesions and lesion-adjacent tissues was significantly associated with the metacommunities identified within carcinoma. Shown are mean *P* values from 1,000 iterations of Fisher's exact tests with Monte Carlo simulation (10,000 replicates). (**b**) Percentages of change between metacommunities from lesion-adjacent mucosae to lesions within each clinicopathologic stage of tumours. LGDP, colorectal polyps with low-grade dysplasia (*n*=39); HGDP, colorectal polyps with high-grade dysplasia (*n*=13); ECRC, early-stage CRC (*n*=26); LCRC, late-stage CRC (*n*=26). (**c**) Significances of fold change in metacommunity markers, as estimated by paired Mann–Whitney *U*-tests, were greatest at early-stage CRC.

The availability of paired samples allowed us to investigate how the microbiome changed at colorectal lesions when compared with adjacent mucosae at different stages. Although the proportion of discordant metacommunities between tumour and tumour-adjacent mucosae were similar in adenoma (36%) and carcinoma (39%) samples, we observed significant patterns of change in community configurations specifically among cancerous mucosae (Fig. 3a; Supplementary Data 6). Remarkably, the sampling of metacommunity E at lesion-adjacent mucosae was almost always accompanied by sampling of the same metacommunity at lesions (92%). The discordances in metacommunity D were mainly explained by the sampling of metacommunity E at lesions relative to lesion-adjacent tissues (69%) (Fig. 3b; see Supplementary Fig. 3 for metacommunity pairs across individuals). Using paired Wilcoxon's signed-rank test, we found no statistical differences in inverse Simpson's diversity index (ISDI) between lesion-adjacent mucosae and lesions (*P*=0.804 in adenoma group; *P*=0.158 in carcinoma group). Nevertheless, there was a significant increase in diversity within carcinomas as compared with adenomas (false discovery rate (FDR)=0.0386).

Using all microbiome parameters described in Fig. 1, we tested whether there were any differences among the subset of individuals with concordant community types between lesions and lesion-adjacent mucosae. Among the matched samples with concordant metacommunity D, the relative abundance of taxa that were classified to Human Oral Microbiome database was moderately higher in lesions than lesion-adjacent tissues (*P*=0.0361; FDR=0.239). This difference was also reflected as an increase in ISDI for lesions (*P*=0.0289; FDR=0.239). By contrast, among samples with matched metacommunity E, there was a moderate decrease in diversity as well as increase in dysbiosis indexes for lesions as compared with lesion-adjacent tissues (ISDI: *P*=0.0479, FDR=0.239; MCPI: *P*=0.0105, FDR=0.210). As for other metacommunities, no difference was found between lesion and lesion-adjacent tissues.

To examine changes in bacterial markers across disease stages, we calculated the fold change of each metacommunity marker relative to lesion-adjacent mucosae. In early-stage CRC, *Fusobacterium, Parvimonas, Gemella* and *Leptotrichia* were most significantly enriched (Fig. 3c), which was accompanied by significant losses of *Bacteroides* and *Blautia*, *F. prausnitzii*, *Sutterella*, *Collinsella aerofaciens* and *Alistipes putredinis*. Neither of these changes was significant in pathological stages of adenoma as well as late-stage CRC (Fig. 3c).

### Interactions of microbial taxa in disease states

**Figure 4:**
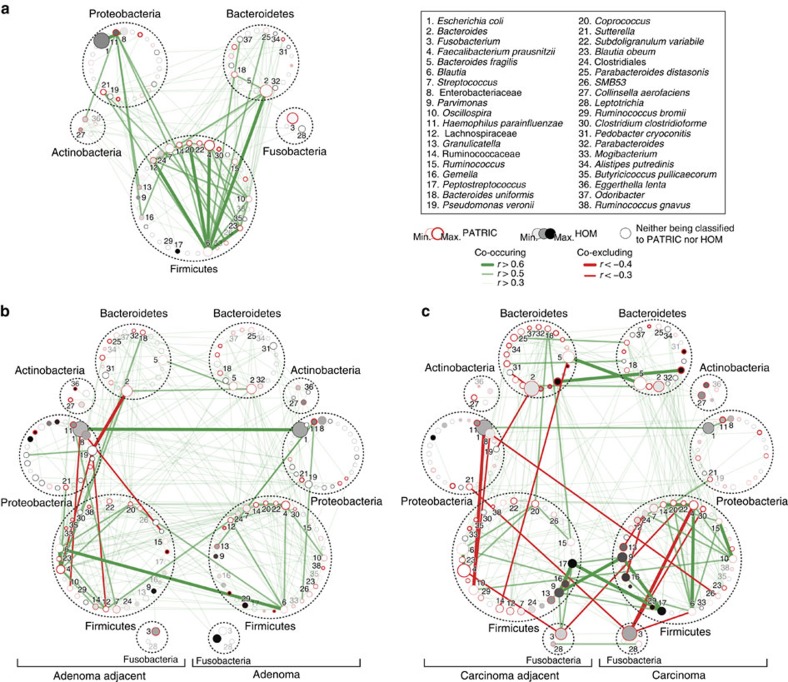
Microbial community ecology at mucosal interface are different across stages of colorectal carcinogenesis. (**a**–**c**) Correlation network of taxonomic partners in: (**a**) normal (*n*=61), (**b**) adenomatous polyps (*n*=52) and (**c**) cancerous mucosae (*n*=52). Correlation coefficients were estimated and corrected for compositional effects using the SparCC algorithm. A subset of correlations with strengths of at least 0.3 was selected for visualization. Node size represents mean taxon abundance in each mucosal phenotype; metacommunity markers are denoted by node numbers accordingly. Taxa that are classified as members of the same bacterial phylum are encircled by dashed lines.

We next inferred all pairwise taxonomic correlations within and/or between normal control, lesion and lesion-adjacent mucosae, using the SparCC algorithm^22^. After iteratively correcting for spurious correlation coefficients and controlling for false discovery rates, we demonstrated that the distribution of taxonomic correlations were significantly different across disease stages (Fig. 4; Supplementary Fig. 4). Among taxa colonizing the normal control mucosae, we found the highest number of significant positive correlations with strengths of 0.5 or above (mean *q*<0.01; Fig. 4a). Interestingly, trans-phylum relationships with strengths of 0.5 or above were less common in disease states than normal colonic mucosae (Fig. 4b,c; see Supplementary Data 7 for the complete list of correlation coefficients with FDR<0.05). Members of the Firmicutes were more likely to form strong co-occurring relationships with one another in normal colonic mucosae than lesions and lesion-adjacent samples. These results indicate that members of the gut microbiota can form niche-specific relationships, which may be a response to an altered colonic mucosal microenvironment or could be one of the reasons for the disease state.

Our network analysis identified significant interactions among several prominent taxonomic members (Fig. 4; Supplementary Data 7). For example, *Parvimonas* and *Peptostreptococcus,* which are members of the oral microbiota, formed one of the strongest positive relationships exclusively within carcinoma and carcinoma-adjacent mucosae. Although *Fusobacterium* was positively related to the oral members of the Firmicutes, the strengths were relatively weak. Nevertheless, the occurrence of *Fusobacterium* was specific to carcinomas as indicated by relatively weak correlation between carcinoma-adjacent mucosae and carcinomas. This was in contrast to the occurrences of *Parvimonas* and *Peptostreptococcus*, which showed strong correlations between carcinoma-adjacent mucosae and carcinomas (Fig. 4c). We also identified several negative relationships of *Fusobacterium* with other taxa, including *Subdoligranulum variabile*, *F. prausnitzii*, *Blautia*, *Clostridium clostridioforme*, and *Sutterella* within and between carcinomas and carcinoma-adjacent mucosae. Among members of the gut commensals, the positive relationship between *F. prausnitzii* and *Blautia* was among the strongest of the Firmicutes in normal control and paired cancerous mucosae. Despite a weaker positive association within and between paired adenoma samples, *F. prausnitzii* exhibited a progressively stronger positive association with members of the Ruminococcaceae toward carcinogenesis. Conversely, the co-occurrence of *Blautia* and *Bacteroides* was remarkably stronger in normal mucosae but weakened with tumour development. Though *E. coli* and members of the Enterobacteriaceae were among the most abundant in paired adenoma samples, their co-occurrence relationship was weaker in paired carcinomas. Besides, *Pseudomonas veronii* correlated positively with low-abundance taxa such as *Massilia*, *Pedobacter cryoconitis*, and members of the Sphingomonadaceae and Erythrobacteraceae, and negatively with *Bacteroides*, *F. prausnitzii* and members of the Lachnospiraceae.

To validate our correlation analyses in independent cohorts, we performed Fisher's exact tests on the total number of significant positive and negative taxonomic relationships that had false discovery rates of 0.25 or less between two studies in comparison. The directions of taxonomic correlations were significantly concordant between our discovery cohort and the two studies (*P*<1.0 × 10^−35^ for both Kostic *et al.*^7^ and Zeller *et al.*^20^ data sets). We also subjected the concordant taxonomic relationships to multiple linear regression analysis to show that the strengths of correlations are significantly supported by the two studies (Supplementary Fig. 5).

## Discussion

Inter-individual variations in tumour-associated mucosal microbiome have posed a long-standing challenge for deciphering microbial signatures implicated in colorectal tumorigenesis. In this study, we demonstrate that as colorectal neoplasm progresses along the adenoma-carcinoma sequence, mucosal microbial communities can establish micro-ecosystems of their own, giving rise to metacommunities of specific structure with functional features that can be predicted (Supplementary Figs 6–8). Although a myriad of factors, such as lifestyle and dietary habits, could contribute to CRC, our systematic analysis highlighted the importance of microbial consortia as a potential player in colorectal tumour development. In this regard, the rediscovery of CRC-specific enrichment of Fusobacterium^7^,^8^,^9^ and *B. fragilis*^23^ and the identification of novel CRC-associated candidates, such as *Gemella*, *Peptostreptococcus* and *Parvimonas*, expands the current scope of bacterial involvement in CRC development. In particular, *Gemella*, *Peptostreptococcus* and *Parvimonas* along with other microbes of oral origin formed a strong symbiotic network, which characterized the CRC-associated metacommunity E. Future studies on their potential oncogenic functions using murine models of CRC will delineate whether these candidates are drivers or passengers in colorectal tumorigenesis.

A unique feature of our experimental design is the sampling of mucosa near the site of a lesion at distinct stages of colorectal neoplasia. With this approach in mind, we have illustrated patterns of discordances in metacommunities between lesions and lesion-adjacent mucosae (Fig. 3b). A novel aspect of CRC pathogenesis that has been recently described is the association of biofilm-forming bacterial communities and their capacity to modulate cancer metabolism^24^,^25^,^26^. Thus, sub-networks of co-occurring and co-excluding microbes at and around neoplastic sites may reflect disease-specific colonic microenvironment (Fig. 4). In particular, we have identified co-exclusive relationships between members of Proteobacteria and Firmicutes in adenoma-adjacent samples. Such changes persisted in carcinoma-adjacent samples, implying that a substantial degree of dysbiosis may have already occurred in the greater colonic environment in tumour-bearing colons. This has a major implication as many gut microbiome studies were based on stool samples, which may reflect the disease state but possibly not the tumour microenvironment.

Our study identified alterations of taxonomic relationships at trans-phylum levels in tumours and tumour-adjacent mucosae. These could be a response to altered host cellular processes, such as energy metabolism and inflammation, at tumour niches. For example, dietary carbohydrate can promote intestinal epithelial cell proliferation^4^ and has been associated with incidences of CRC^27^,^28^. Inflammation or colitis-associated niche may also favour the growth of specific bacterial populations that could elicit oncogenesis^29^,^30^,^31^,^32^. In adenomatous lesions, the enrichments of *E. coli* and *P. veronii* are intriguing, raising the possibility of bacteria-triggered mutagenesis (see Supplementary Data 8 for detection of *pks* genomic islands for *E. coli*) as well as host-microbiome lateral gene transfer^33^,^34^, both of which may drive transformation of otherwise benign colonocytes by influencing genomic stability. Similarly, predicted enrichments in functional potentials for xenobiotics metabolism^35^, utilization of polyamines^36^, and degradation of polycyclic aromatic compounds^37^ in metacommunities C and E (Supplementary Fig. 6) may suggest an increased susceptibility of colonocytes for pro-tumorigenic bacterial metabolites. Furthermore, the association of bacterial peptidoglycan biosynthesis pathways with metacommunity E (Supplementary Fig. 6) may modulate local inflammation in evolving neoplasms^38^ by enhancing intestinal cell permeability^30^, which may allow for a vicious cycle of tumour-potentiating activities of co-occurring invasive bacterial species. However, given the hypothetical nature and potential database biases in metagenome imputation, it remains to be determined whether or how such functional traits of gut microbial communities affect host cells during colon tumorigenesis.

An important issue that could not be directly addressed by our study is the identification of adenoma-associated metacommunities that are predictive of cancer progression. On the other hand, we have identified bacterial operational taxonomic units (OTUs) with progressively increasing abundance, such as *B. fragilis* and *Granulicatella* (Supplementary Fig. 9), in the adenoma-carcinoma sequence. *B. fragilis* is known to induce signal transducer and activator of transcription 3 and Th17-dependent pathway in colitis-associated CRC (see Supplementary Data 8 for detection of *bft* genes from enterotoxigenic *B. fragilis*)^37^ whereas the abundance of *Granulicatella adiacens* in saliva is associated with chronic pancreatitis and pancreatic cancer^39^. These bacterial candidates will require functional validations to assess their prognostic values for tumour recurrence in polypectomized adenoma patients in future prospective studies. Another limitation of our study is that mucosa-associated microbiota could be altered by bowel cleansing preparation and reagents^40^. However, this is inevitable given the necessary procedure for sample acquisition.

Our study marks an additional step towards defining mucosal community configurations in colorectal tumorigenesis. Perhaps the most practically challenging step is the temporal association of metacommunities with pre-onset monitoring and post-manifestation follow-up of diseases. Future genomic analyses interrogating the cross-talk between subtypes of immune cell populations, host cell epigenomes and microbial consortia will be essential to define the multifaceted roles of gut microbiome in human health and diseases.

## Methods

### Patient recruitment and informed consent

We enroled individuals who had undergone standardized colonoscopic examinations at the Prince of Wales Hospital of the Chinese University of Hong Kong and the First Affiliated Hospital of Sun Yat-Sen University in Guangzhou between March 2011 and January 2014. Mucosal biopsies were obtained from a total of 160 individuals with tumour-free colon (*n*=61), with confirmed histology of colorectal polyps (*n*=47), or with invasive adenocarcinomas (*n*=52). We also recruited an independent cohort of 116 individuals of which 25 subjects had normal colons, 41 subjects had colorectal adenomas, and 50 subjects were diagnosed with CRC, from the Beijing Military General Hospital. Written informed consents were obtained from subjects or their authorized representatives. Samples originating from Hong Kong were collected as part of a screening cohort, which has been previously described^41^,^42^,^43^. Eligibility criteria for colonoscopy included: (1) age 50–70 years; (2) absence of existing or previous CRC symptoms, such as haematochezia, tarry stool, change in bowel habit in the past 4 weeks, or a weight loss of >5 kg in the past 6 months and (3) not having received any CRC screening tests in the past 5 years. Samples originating from Chinese populations in Guangzhou and Beijing were collected through routine colonoscopy services for conventional indications, including (1) CRC symptoms such as haematochezia, tarry stool, change in bowel habit or weight loss; (2) positive faecal occult blood; (3) abnormal imaging such as barium enema, computed tomography, magnetic resonance imaging or positron emission tomography. The exclusion criteria for colonoscopy included: (1) personal history of CRC, inflammatory bowel disease, prosthetic heart valve or vascular graft surgery and (2) the presence of medical disorders, which were contraindications for colonoscopy.

Polyethylene glycol powders (Klean-Prep, Helsinn Birex Pharmaceuticals, Ireland) were mixed with 4 l of cathartic suspension for use as standard bowel preparation regime among all participants. Air insufflation was used for all procedures, which were performed by experienced colonoscopists in the endoscopy centres of each hospital in this study; we strictly aimed for caecal intubation and a withdrawal time of more than 6 min according to the current quality indicators for colonoscopy. Multiple mucosal biopsies were taken from each colorectal tumour with the greatest dimension of at least 0.5 cm and subsequently evaluated by H&E staining at the pathology suite. Biopsies were snap-frozen in cryovial immediately after polypectomy and stored at –80 °C until DNA extraction. Adjacent normal tissues were taken at least 4 cm away from lesions. Colorectal mucosae were obtained using cold biopsy forceps separately for lesions and lesions-adjacent tissues to avoid cross-contamination between samples. The histopathology reports were made according to the checklist recommended by the College of American Pathologists (3.1.0.0). Control biopsy samples were provided by individuals who had no lesion detected during colonoscopy. Although the biopsies originated in various anatomical regions throughout the caecum, colon and rectum, we observed no significant biogeographical bias in metacommunities sampled (Supplementary Data 1). Any nucleic acid or remaining biopsy samples from participants who withdrew consent after endoscopic examinations were destroyed. As enroled subjects had highly stratified medical records, we tested whether the observed inter-individual differences in mucosal microbiome profiles were due to potentially confounding effects of subject demographics and laboratory-proven clinical diagnoses (Supplementary Fig. 10; Supplementary Data 1,9 and 10). The study conformed to the ethical principles outlined by the Declaration of Helsinki and was approved by the Institutional Review Boards of the Chinese University of Hong Kong, the Sun Yat-Sen University and the Beijing Military General Hospital.

### Preparation of DNA amplicon library

For optimal isolation of bacterial DNA^44^, mucosal biopsies were disrupted by bead-beating on digestion in enzymatic cocktail of mutanolysin and lysozyme (Sigma) before extraction and purification by QIAamp DNA Mini Kit, and quantification by Agilent 2100 Bioanalyzer. Amplicon library for unidirectional sequencing (Lib-L) on the 454 GS FLX+ Titanium platform was constructed using fusion primers ligated by Roche adaptor sequences, Multiplex Identifier (MID) tags, library keys, and template-specific sequences (27F-800R) targeted across the hypervariable regions 1–4 of 16S rRNA genes. DNA library was subsequently purified (AMPure XP), quantified (Quant-iT PicoGreen dsDNA Assay Kit), and subjected to quality control by cleanup of short amplicon fragments according to manufacturer's instructions.

### Sequence curation pipeline

Quality control of sequencing read was implemented as described in mothur software suite^16^. Flowgrams were pre-processed by retaining all that had fewer than two mismatches and one or zero mismatch to the primer and barcode, respectively, and trimmed to 1,050 flows before the removal of pyrosequencing noise using the PyroNoise algorithm^45^. The de-noised reads were demultiplexed by removing sample-specific barcodes, further processed by removing any that had homopolymers longer than 10 nucleotide bases and/or had an ambiguous base call, and aligned against the non-redundant SILVA database (version 119) using the NAST algorithm^46^. Any sequence that failed to align with the V1-4 region as predicted by the primer set was discarded; the remaining sequences were trimmed to the same alignment coordinates over which they fully overlapped, clustered with more abundant sequences by a maximum difference of five nucleotide bases^17^, and detected for the presence of chimeras by *de novo* UChime^47^. The resulting sequences were classified against the Greengenes database (version 13.8) and annotated with deepest level taxa represented by pseudo-bootstrap confidence scores of at least 80% averaged over 1,000 iterations of the naive Bayesian classifier^48^. Any sequences that were classified as either being originated from eukarya, archaea, mitochondria, chloroplasts or unknown kingdoms, were removed. The annotated sequences were assigned to phylotypes according to their consensus taxonomy with which at least 80% of the sequences agreed (see Supplementary Fig. 11 for taxonomic breakdown at class level). The final sequence count table contained 8,197±4,471 (mean±s.d.) reads per sample with a minimum and maximum read length of 450 and 623 nucleotide bases, and was rarefied at 1,000 reads per sample to reduce the effects of variable sequencing depths on downstream analyses (Supplementary Fig. 12).

### Determination of optimal microbial community clusters

Effects of binning rare phylotypes by their total relative abundance in the rarefied data set containing 592 taxa were assessed to determine whether two general methods agreed over a certain range of rarity thresholds in detecting optimal numbers of cluster: PAM^49^ and DMM modelling^18^. Procrustes analysis of truncated data sets, which were generated by applying rarity cutoffs of up to 10%, consistently demonstrated minimal cutoff-by-cutoff variations to the results of the non-metric multidimensional scaling: *R*=0.994±0.005 (mean±s.d.)^50^. When changing rarity definitions between 0–1% for model fitting, the total number of reads per sample were preserved by grouping rare phylotypes. At around 0.1% rarity cutoff, we observed that a core list of 99 taxa were sufficient to detect the most comprehensive number of microbial community clusters as identified by both Calinski–Harabasz index and the Laplace approximation to the model evidence. When changing rarity definitions above 0.1% at increments, the robustness of PAM-based approach varied in contrast to DMM-based approach (Supplementary Fig. 1a).

### Prediction of metabolic potentials

Sequences from post-quality control were assembled into reference-free OTUs at 3% distance using the average neighbour algorithm as implemented within mothur^16^ (see Supplementary Fig. 13 for number of shared OTUs between disease states). Consensus taxonomy with a confidence score of at least 80% was generated for each OTU and the OTU count table was picked against the Greengenes reference OTU identifiers (version 13.5) for use in the two-step functional inference pipeline PICRUSt (ref. 51). The PICRUSt uses precomputed gene copy numbers for KEGG Orthologous families based on finished bacterial genomes available in the Integrated Microbial Genome database to predict the gene family content for all microorganisms represented by the 16S-based Greengenes phylogeny, including OTUs with unknown gene content for which previously sequenced evolutionary relatives are available. The input OTU table was normalized by the predicted 16S rRNA gene copy numbers to estimate the true organismal abundances before the multiplication of the pre-calculated set of gene family counts for each taxon by the abundance of that OTU. The resulting metagenomic copy number table consisted of 6,909 KEGG Orthologous entries and served as input data in the HUMAnN pipeline that outputs the relative abundances of known microbial metabolic modules and pathways as defined by KEGG for each sample based on the user-provided table of gene family counts^52^. A total of 118 and 169 KEGG functional modules and pathways, respectively, were derived from the predicted metagenomic data. See Supplementary Figs 6–8 and Supplementary Data 11 and 12 for results of differential abundance analyses on gene families using the LEfSe algorithm.

### Correlation network inferred by phylogenetic marker genes

The rarefied data set containing 99 phylotypes, which were previously selected for the detection of microbial community clusters through DMM modelling, was subjected to compositionality data analysis using the SparCC algorithm, which is known for its robustness to the compositional effects that are influenced by the diversity and sparsity of correlation in human microbiome data sets^22^. Taxon–taxon correlation coefficients were estimated as the average of 20 inference iterations refined by 100 exclusion iterations with the default strength threshold. A total of 10,000 simulated data sets were generated to calculate the corresponding empirical *P* values. This set of iterative procedures were applied separately to normal control, adenoma and carcinoma data sets to infer the basis correlation values within and/or between paired sampling sites. Correlation coefficients with magnitude of 0.3 or above were selected for visualization in Cytoscape (version 3.1.1).

### Definition of microbial community polarization index

Inspired by how one's microbiome profile can be summarized by the Microbial Dysbiosis index (MDI) as an important indicator of disease^53^, we designed a composite index of the MDI to describe how the level of microbial diversity is associated with colonic tumour burden. We calculated the fold change for each representative taxon from a community cluster by dividing the mean abundance in paired samples by that of normal controls and required a marker taxon to have a minimum fold change of 1.5 to be selected as an elemental variable of the MDI. We intended to define the MCPI as a measure of overall dysbiotic shifts that were more characteristic of adenoma over carcinoma, or vice versa (Fig. 1a, top upper panel; Supplementary Fig. 2). The MCPI of sample *j* was computed as follows:


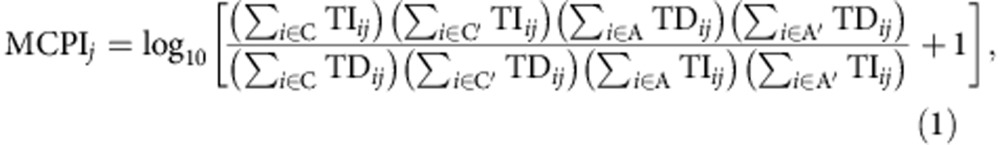


where TI_*ij*_ (or TD_*ij*_) is the abundance of a marker taxon *i* increased (or decreased) in either case of carcinoma, carcinoma-adjacent, adenoma or adenoma-adjacent, which are denoted by C, C′, A and A′, respectively.

### Details of statistical methods

Differential abundance analyses were performed using the LEfSe algorithm to identify significant gene markers that consistently differentiated at least one (or multiple) feature(s) in comparison with the others^54^. The biomarker relevance was ranked according to bootstrapped (*n*=30) logarithmic linear discriminant analysis scores of at least 2. Using the R implementation of Random Forests tenfold cross-validations with 100 iterations^19^, we selected a minimum set of bacterial taxa that maximally discriminated against each mucosal phenotype; the variable importance of a microbial taxon was determined by 100 iterations of the algorithm with 3,000 trees and the default mtry of *p*^1/2^, where *p* is the number of input phylotypes. To evaluate the performance of markers that typify metacommunities against those that are selected by supervised classification on mucosal phenotypes; we constructed LASSO logistic regression models with tenfold repeated internal cross-validations to mitigate the risks of over-fitting train-sets when predicting each test-set^55^,^56^. The data set was partitioned in such a way that each sample was selected exactly once by test-sets for which the prediction scores were generated for use with receiver operating characteristic analysis (Supplementary Fig. 2). Similarly, we subjected our discovery cohort data set to LASSO model training for five-way prediction of metacommunities in independent cohorts (Supplementary Data 5). Furthermore, we performed Kolmogorov–Smirnov tests to assess whether the observed differences in taxonomic relationships are statistically significant between disease states (Supplementary Fig. 4). For associations with categorical and continuous clinical metadata, and confounding factor analyses of microbiome metrics and relative abundance data of metacommunity markers, we applied Fisher's exact tests, Mann–Whitney *U*-tests, and multinomial logistic regression models, where appropriate. Statistical significances of multiple comparisons were corrected by Benjamini–Hochberg step-up procedure.

## Additional information

**Accession codes:** 16S rRNA gene sequences analysed in this study have been deposited in the NCBI SRA database under the BioProject ID: PRJNA280026.

**How to cite this article:** Nakatsu, G. *et al.* Gut mucosal microbiome across stages of colorectal carcinogenesis. *Nat. Commun.* 6:8727 doi: 10.1038/ncomms9727 (2015).

## Supplementary Material

Supplementary Information Supplementary Figures 1-13 and Supplementary Tables 1-2

Supplementary Data 1 Pairwise associations of metacommunities with clinical variables

Supplementary Data 2 Summary of differential abundance analyses on bacterial phylotypes

Supplementary Data 3 Results of Random Forests classification on mucosal phenotypes. Highlighted yellow are the top 18 bacterial phylotypes with highest mean decrease in accuracy selected by 100 iterations of ten-fold cross-validation

Supplementary Data 4 Pairwise comparisons of microbiota metrics between mucosal phenotypes and metacommunities

Supplementary Data 5 Multiclass prediction of metacommunities by LASSO models in independent cohorts

Supplementary Data 6 Pairwise associations of metacommunity concordance or discordance with clinical variables

Supplementary Data 7 Summary of pairwise taxonomic correlations among disease-states

Supplementary Data 8 Results of real-time PCR screening for mucosa-associated *pks* genomic islands of *Escherichia coli* and *bft* genes of enterotoxigenic *Bacteroides fragilis*

Supplementary Data 9 Multinomial logistic regression analysis of bacterial phylotypes on mucosal phenotypes with clinical parameters as co-variates

Supplementary Data 10 Multiple linear regression analysis of bacterial phylotypes on microbiota metrics with clinical parameters as co-variates

Supplementary Data 11 Summary of differential abundance analyses on KEGG modules

Supplementary Data 12 Summary of differential abundance analyses on KEGG pathways
