# Supplementary material for: Gut mucosal microbiome across stages of colorectal carcinogenesis
Source: Nat Commun. 2015 Oct 30;6:8727. doi: 10.1038/ncomms9727 (PMC4640069; doi:10.1038/ncomms9727)
Supplement: Supplementary Information — Supplementary Figures 1-13 and Supplementary Tables 1-2 [file ncomms9727-s1.pdf]

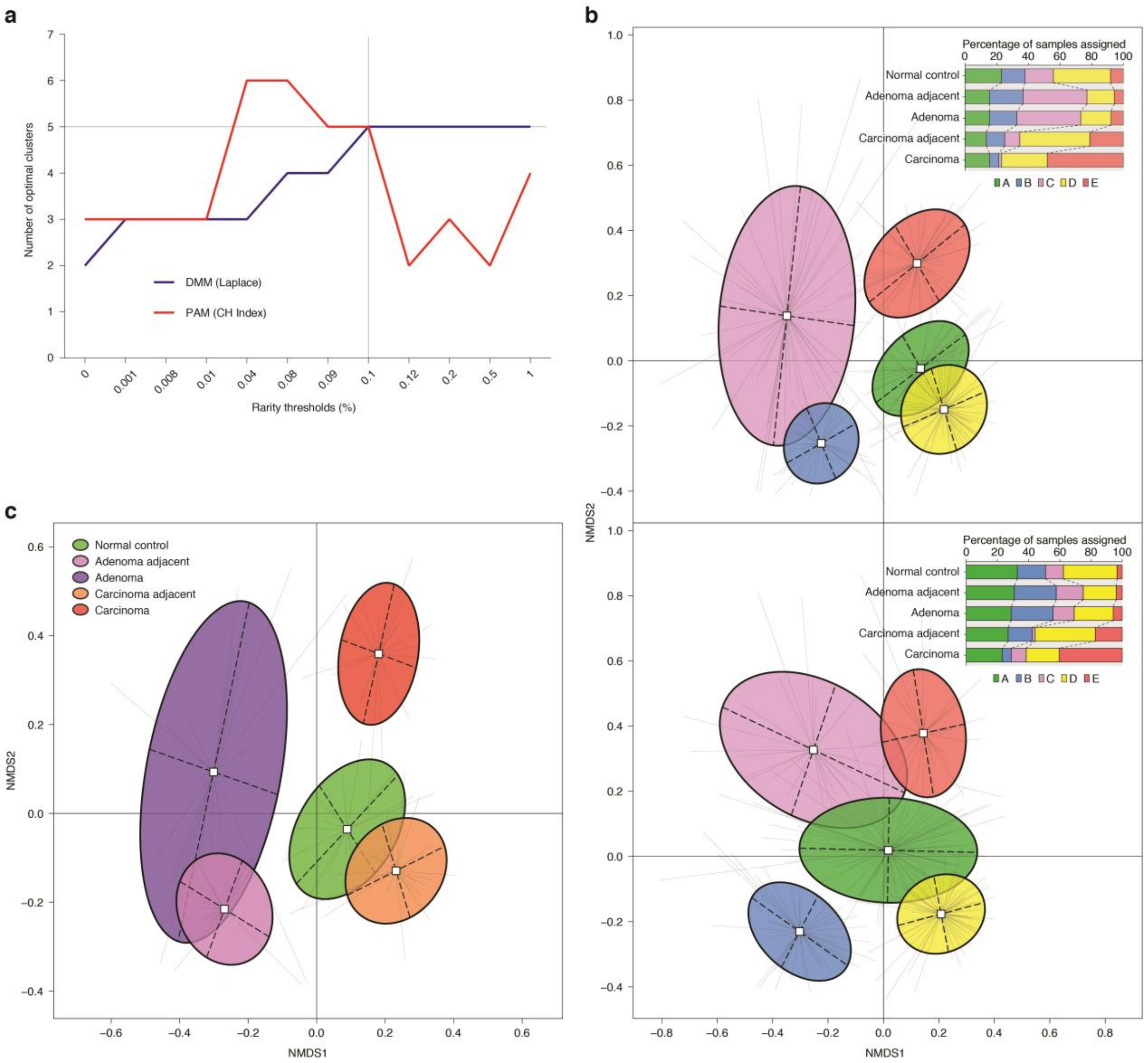

Supplementary Figure 1 | Dimension reduction of metacommunities associated with colorectal carcinogenesis. (**a**) Robustness of two widely used partitioning approaches in response to varying rarity thresholds. (**b**) Comparison of DMM- (top) and PAM-based (bottom) detection of microbial community clusters using non-metric multidimensional scaling (NMDS) of Jensen-Shannon Divergence distance matrices. (**c**) NMDS ordination of representative samples from DMM-based approach using relative abundance profiles of metacommunity markers. Metacommunities are represented by 80% confidence ellipses.

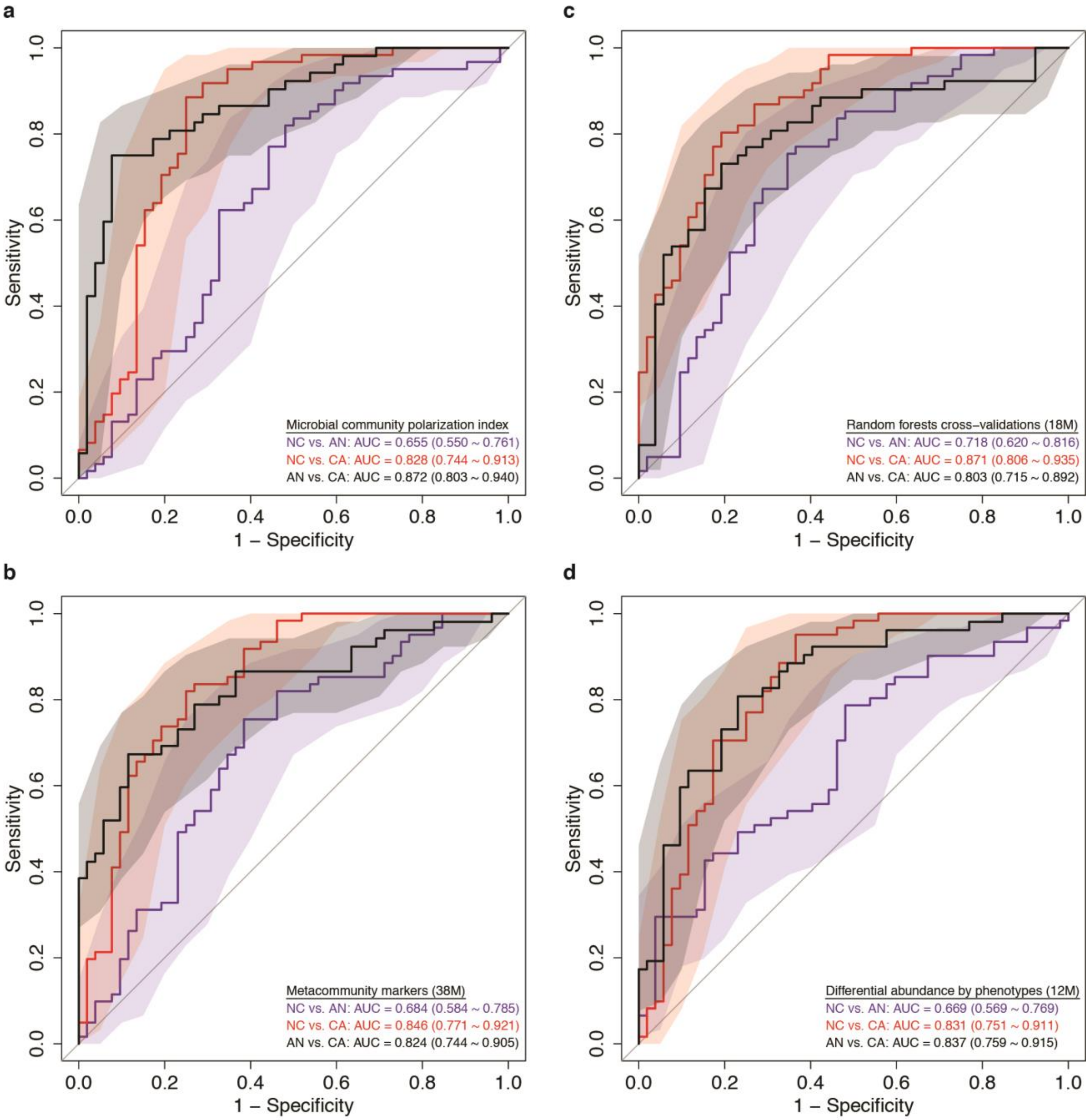

Supplementary Figure 2 | Microbiome-based classification of colorectal tumour statuses. Receiver operating characteristic (ROC) analyses of (**a**) Microbial Community Polarization index (MCPI) and (**b-d**) LASSO classifier performance based on bacterial phylotypes that were identified by (**b**) DMM community typing, (**c**) 100 iterations of the ten-fold Random Forests cross-validations, and (**d**) differential abundance analysis using the LEfSe algorithm. AUC values are shown with 95% confidence intervals (shaded-area). NC, normal control; AN, adenoma; CA, carcinoma.

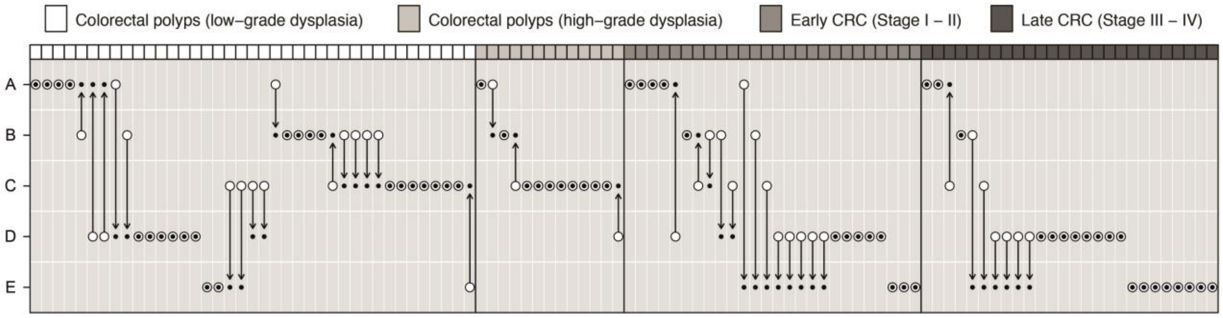

Supplementary Figure 3 | Alterations of mucosal community types at lesions relative to adjacent normal mucosae along the adenoma-carcinoma sequence. Open and closed circles represent lesion-adjacent mucosae and lesions, respectively.

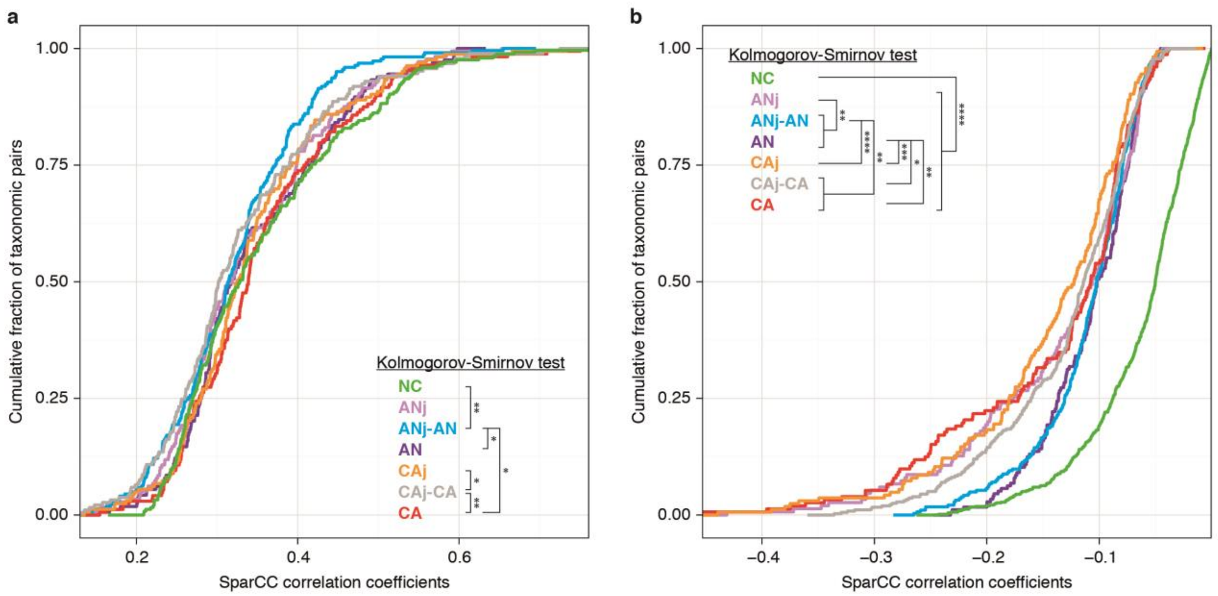

Supplementary Figure 4 | Cumulative distribution functions comparing the differences in taxonomic occurrences between disease-states. Correlation coefficients with statistical significances (FDR < 0.1) were selected for visualization. Distances between two-sample distributions were assessed separately for each group of (**a**) positive and (**b**) negative correlations by Kolmogorov-Smirnov tests. *P*-values were adjusted by BH step-up procedure; * $q < 0.05$; ** $q < 0.01$; *** $q < 0.001$; **** $q < 0.0001$. NC, normal control; ANj, adenoma-adjacent; AN, adenoma; CAj, carcinoma-adjacent; CA, carcinoma.

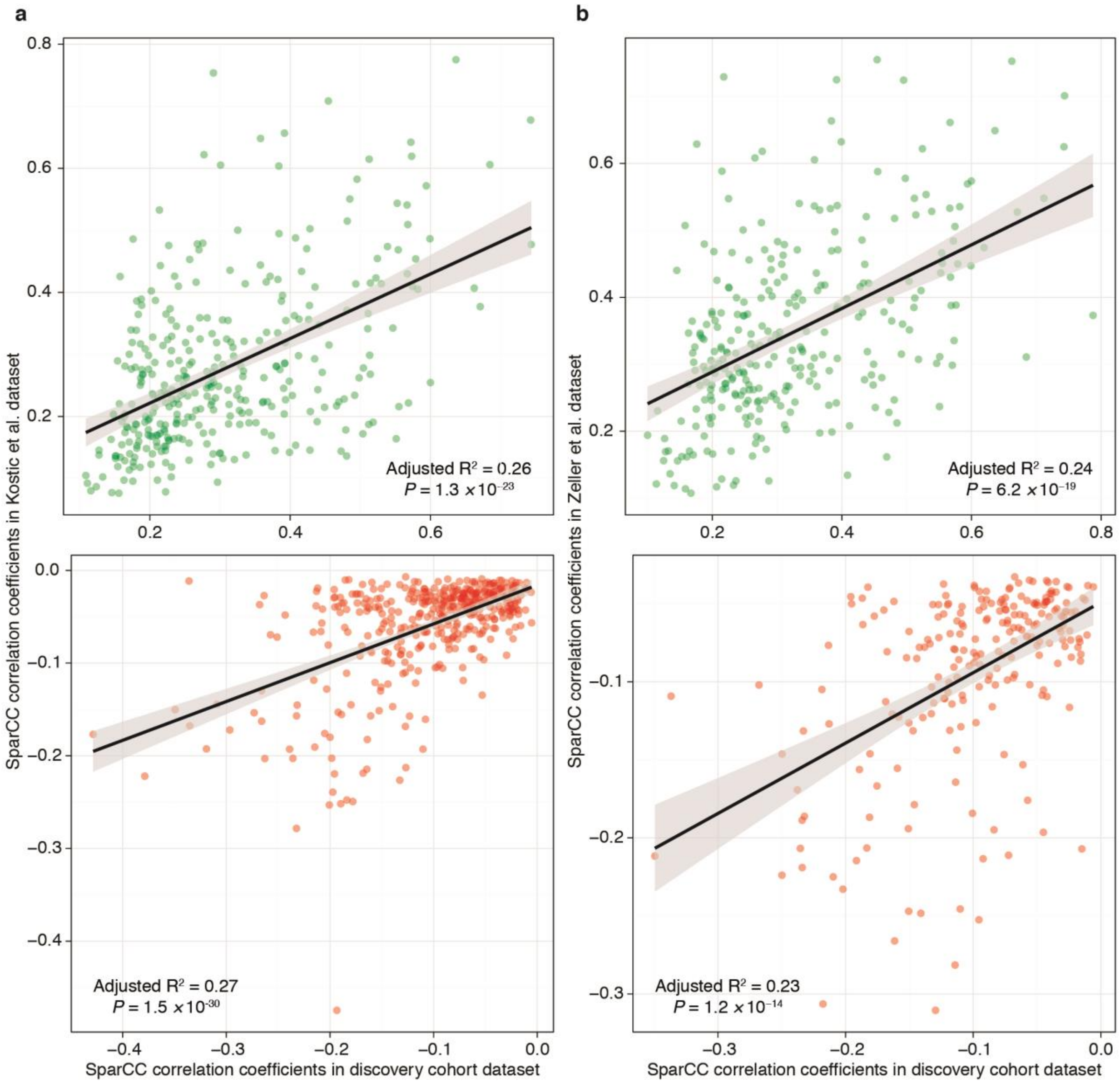

Supplementary Figure 5 | Reproducibility of ecological interactions within cancer-niches. Re-analysis of taxonomic relationships revealed analogous patterns of statistically significant interactions in: (**a**) Kostic et al. dataset, and (**b**) Zeller et al. dataset. Shown are correlation coefficients (green for positive; red for negative) with concordant directions and false discovery rates of 0.25 or less between two studies. Adjusted R^2 and *p*-values are from multiple linear regression analyses.

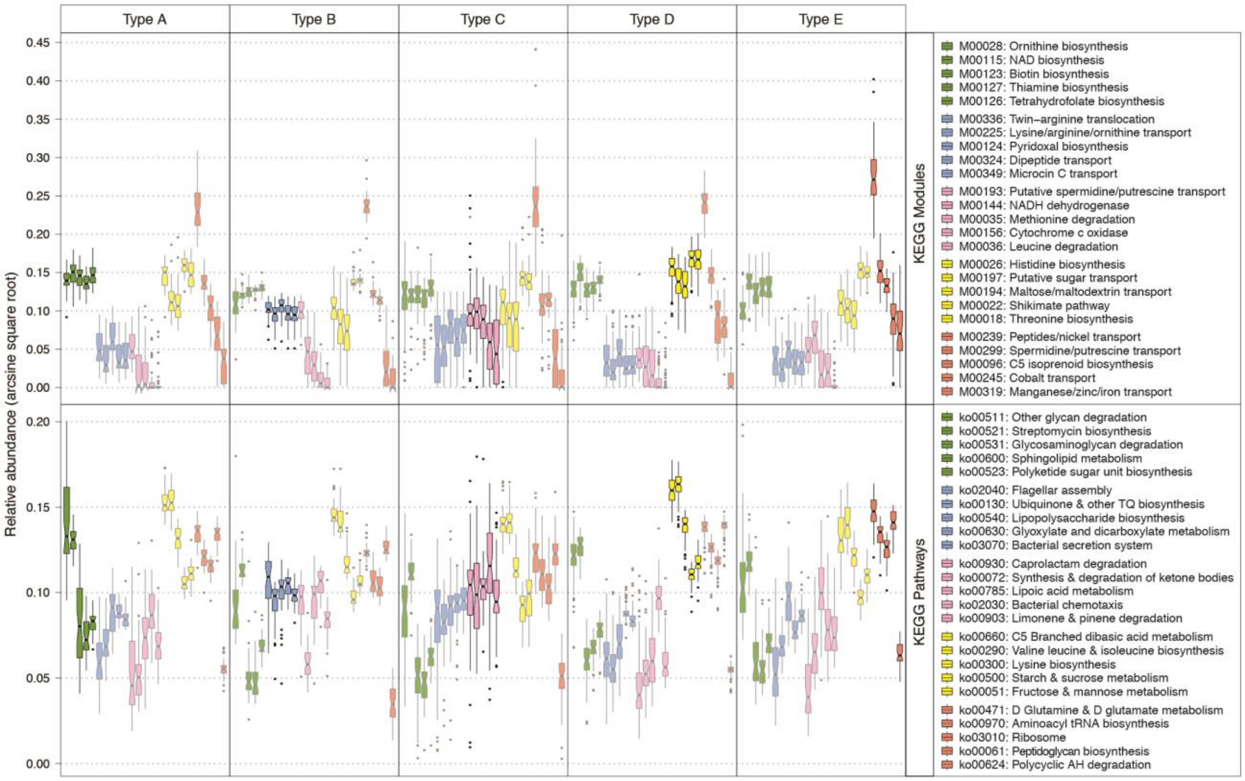

Supplementary Figure 6 | Representative functional modules and pathways that are enriched in metacommunities. Modules or pathways are listed in the descending order of LDA scores from top to bottom in the legend keys and from left to right in the boxplot panels. Gene families are represented by the metacommunity in which they are overrepresented; underrepresented families are greyed out to provide contrasts for visualization. The heights of boxes show the interquartile range (IQR) between the first and third quartiles in which medians are bolded; minimum and maximum values are denoted by whiskers; closed-circles are outliers.

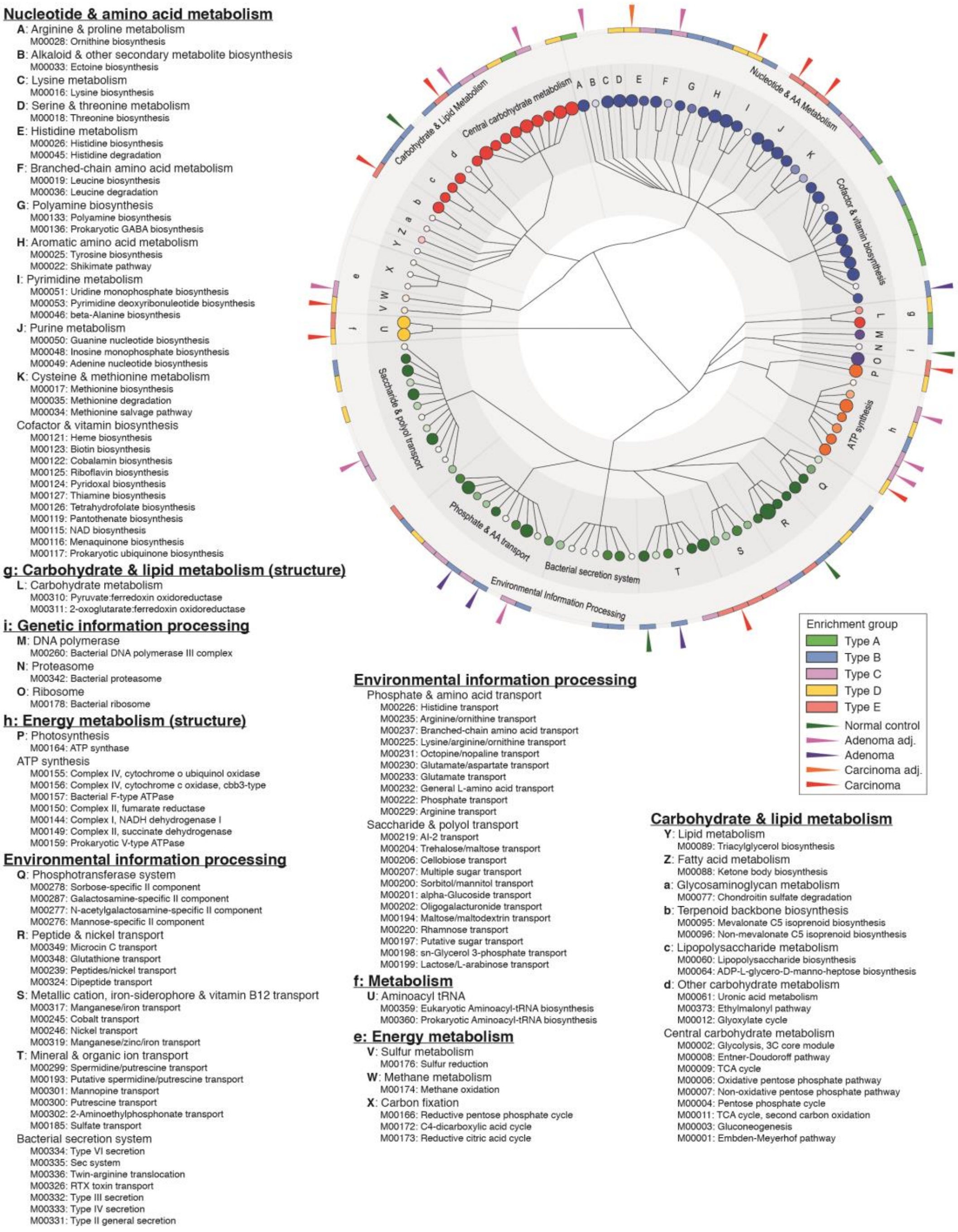

Supplementary Figure 7 | Summary cladogram of differentially abundant KEGG modules imputed. Node size and transparency represent the total relative abundance and prevalence of a module, respectively. Clades and nodes are annotated in a clockwise manner. Functional categories at level 2 of the BRITE module hierarchy are distinguished by respective node colors. Node color intensity is proportional to the coverage of a module. Inner and outer ring indicate differential enrichments of modules based on metacommunity and mucosal phenotypes, respectively.

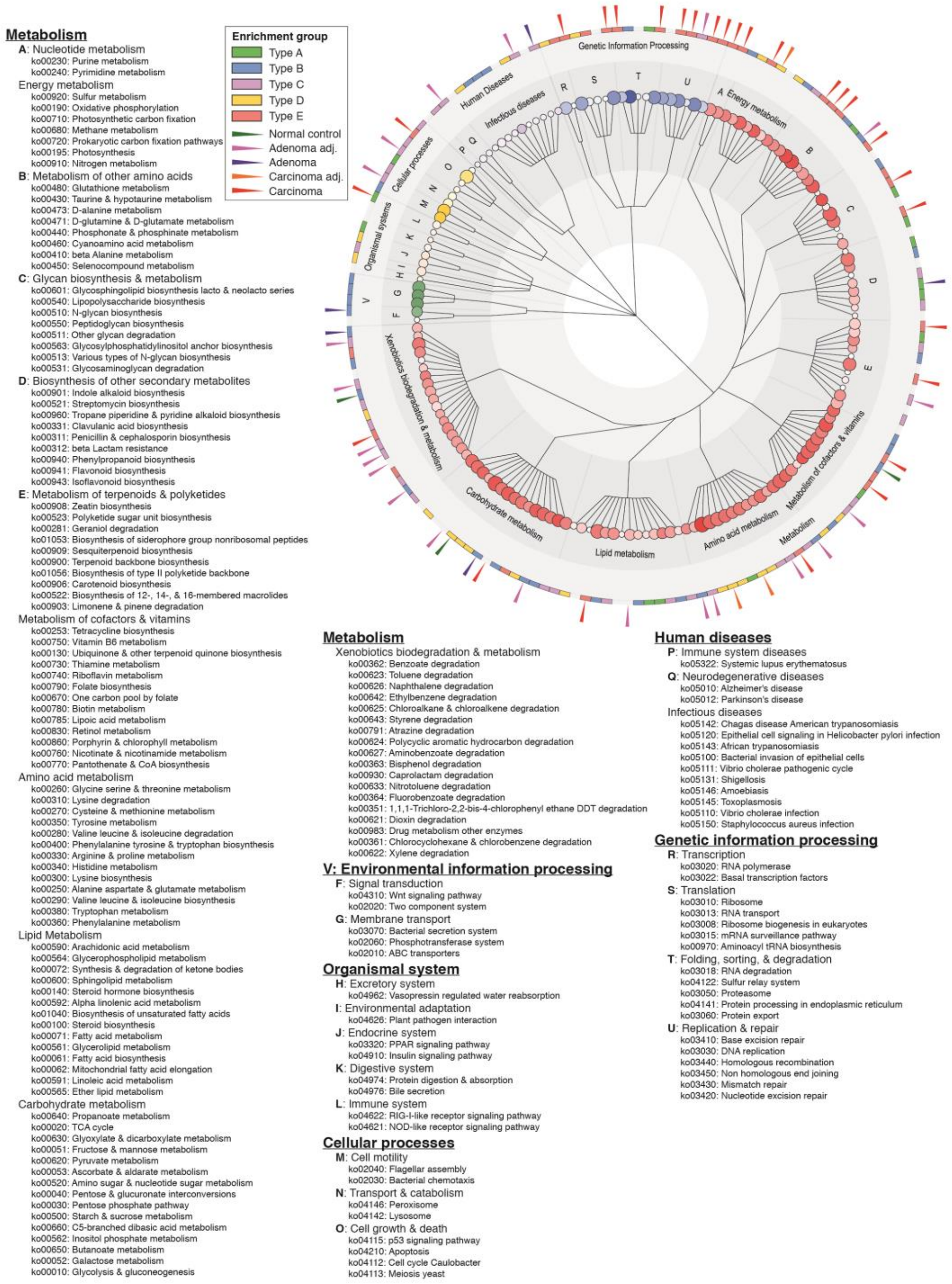

Supplementary Figure 8 | Summary cladogram of differentially abundant KEGG pathways imputed. Node size and transparency represent the total relative abundance and prevalence of a pathway, respectively. Clades and nodes are annotated in a clockwise manner. Functional categories at level 1 of the BRITE pathway hierarchy are distinguished by respective node colors. Inner and outer ring indicate differential enrichments of modules based on metacommunity and mucosal phenotypes, respectively.

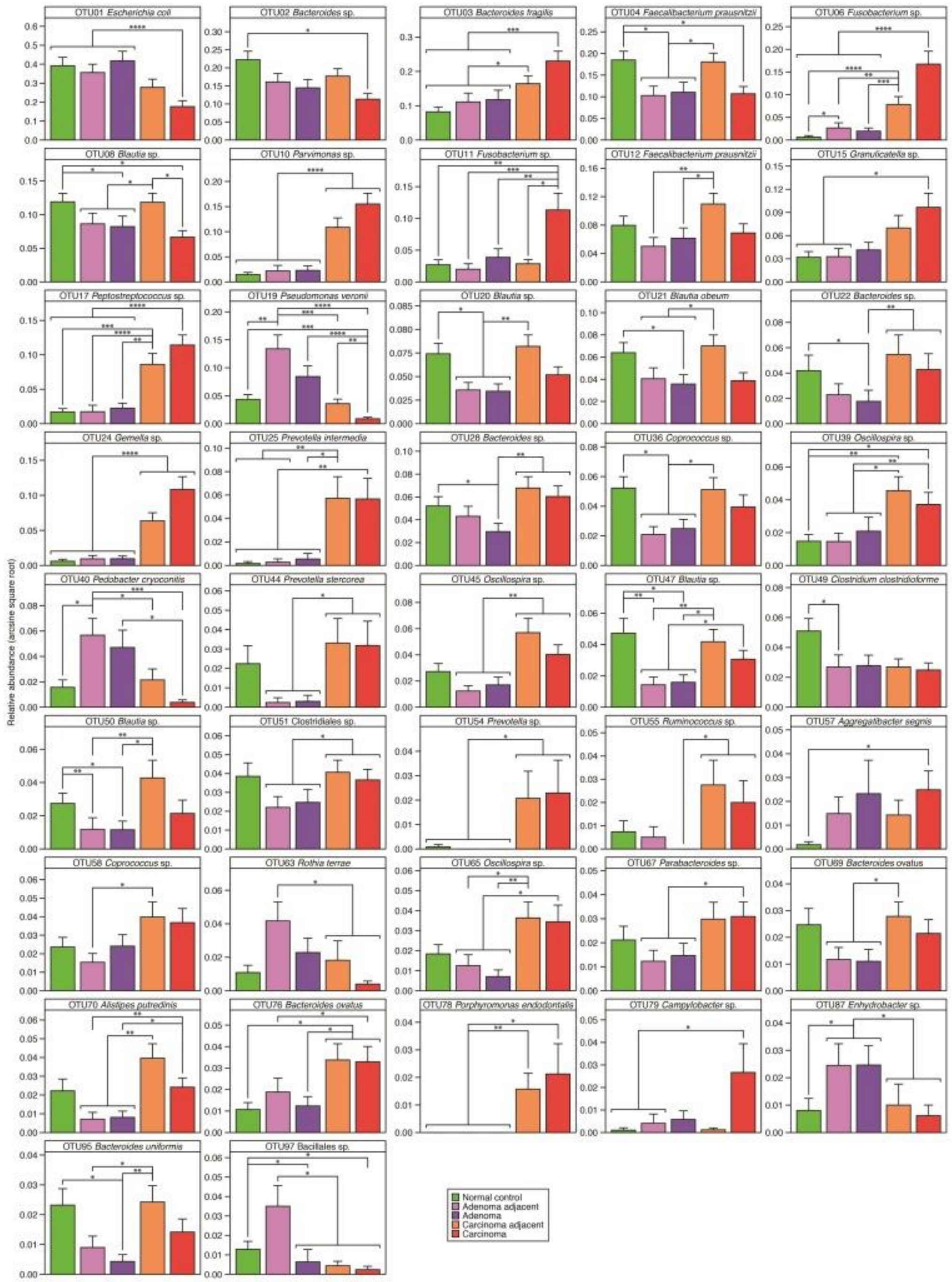

Supplementary Figure 9 | Relative taxonomic abundance of the top 42 operational taxonomic units (OTUs) at 97% identity. OTUs are ranked in the ascending order of the size of total relative abundance after rarefication. Error bars represent standard errors of the means (SEMs). Mann-Whitney U test corrected by BH step-up procedure; * $q < 0.05$; ** $q < 0.01$; *** $q < 0.001$; **** $q < 0.0001$.

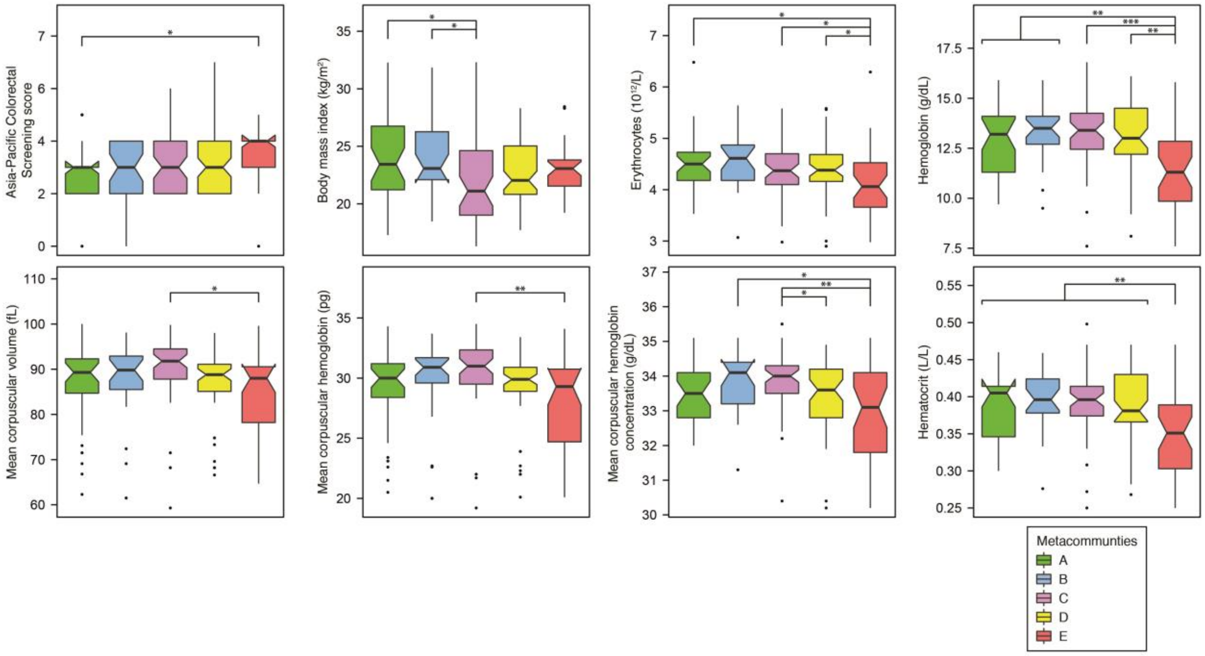

Supplementary Figure 10 | Associations of metacommunities with clinical metadata. The heights of boxes represent the IQR between the first and third quartiles in which medians are bolded; minimum and maximum values are denoted by whiskers; closed-circles are outliers. Mann-Whitney U tests corrected by BH step-up procedure; * $q < 0.05$; ** $q < 0.01$; *** $q < 0.001$.

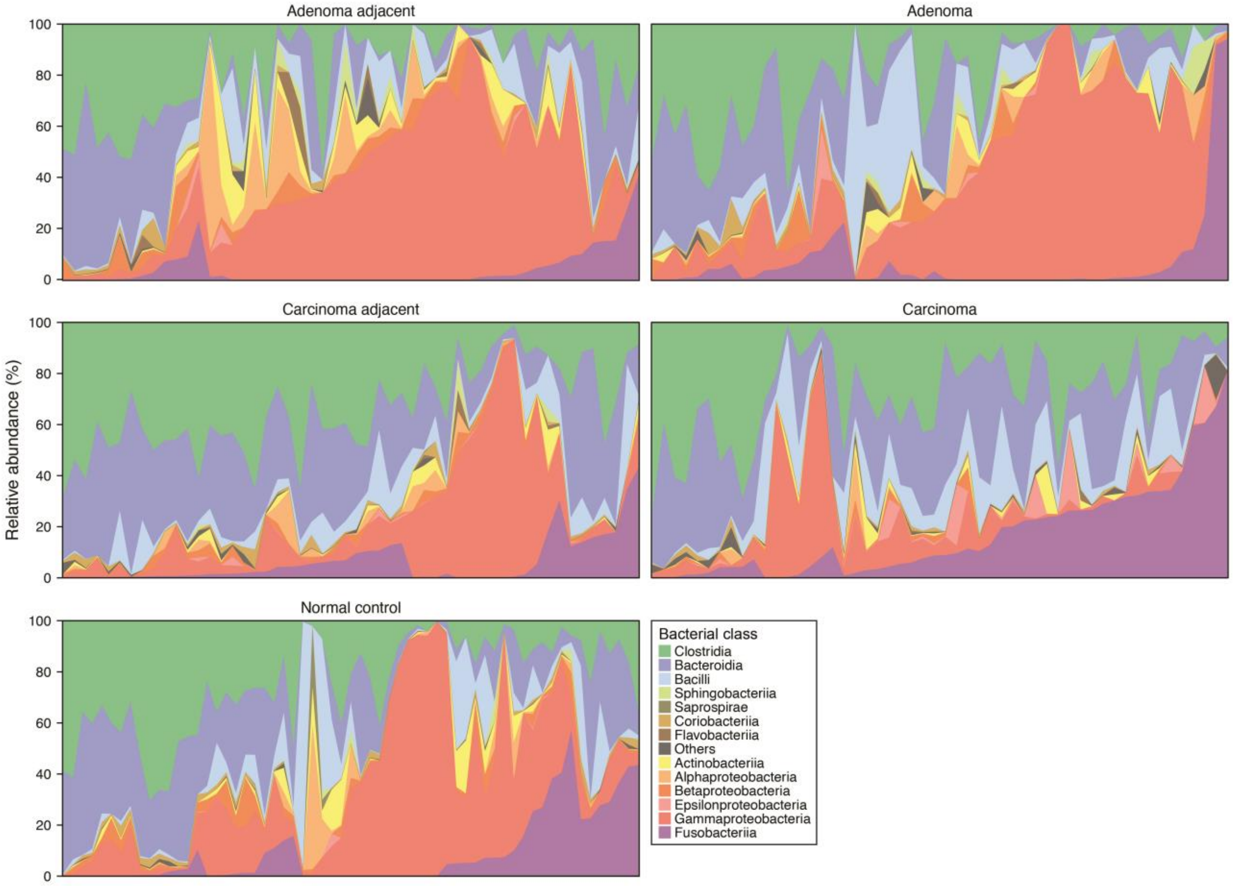

Supplementary Figure 11 | Breakdown of sequencing reads at bacterial class-level. Areas represent microbiome profiles of mucosal biopsies from normal (*n* = 61), adenoma-affected (*n* = 47), carcinoma-affected (*n* = 52) colon.

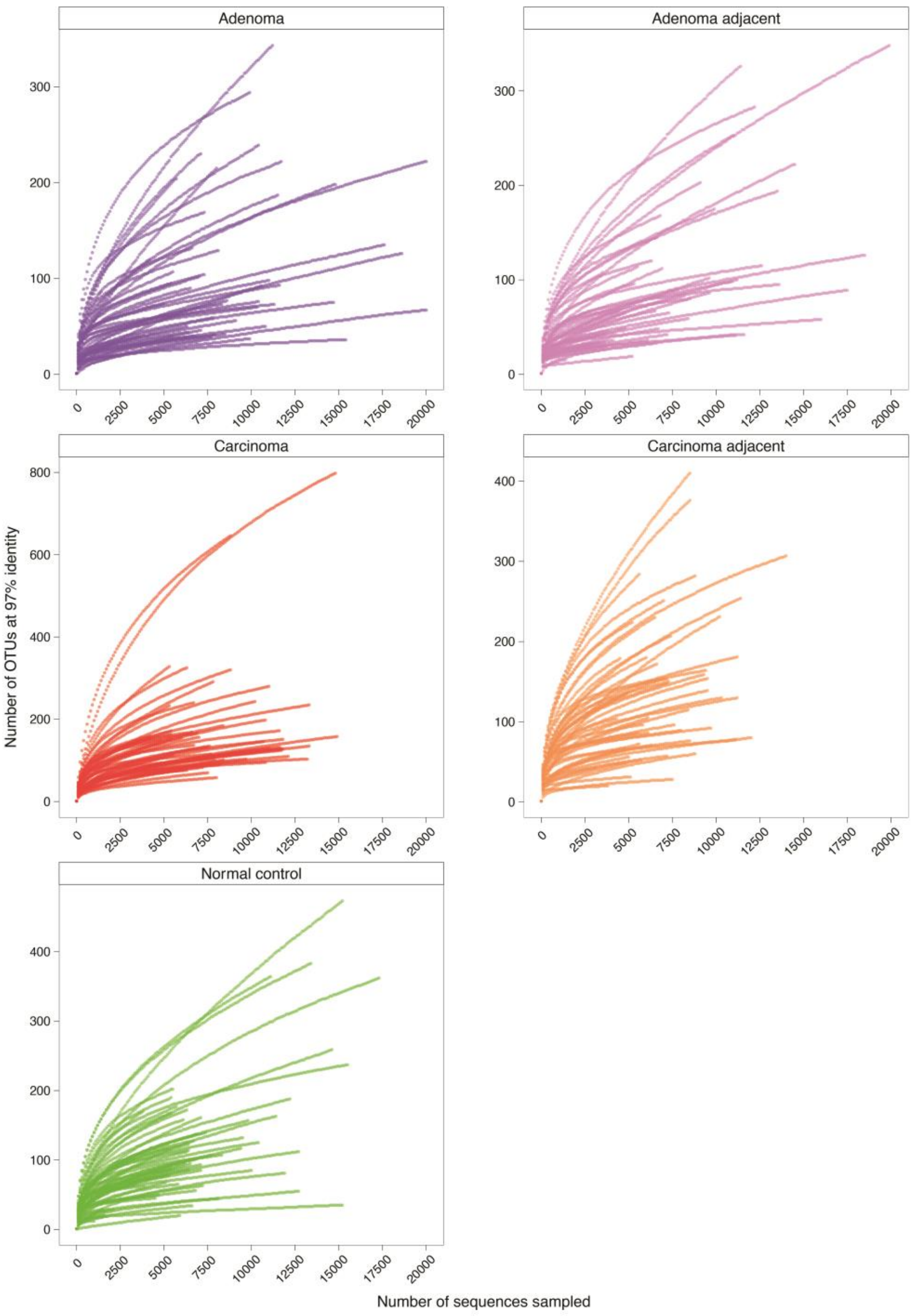

Supplementary Figure 12 | Comparisons of OTU sampling depths among biopsy phenotypes. Rarefaction curves describe the number of 97% OTUs detected as sequencing effort increases.

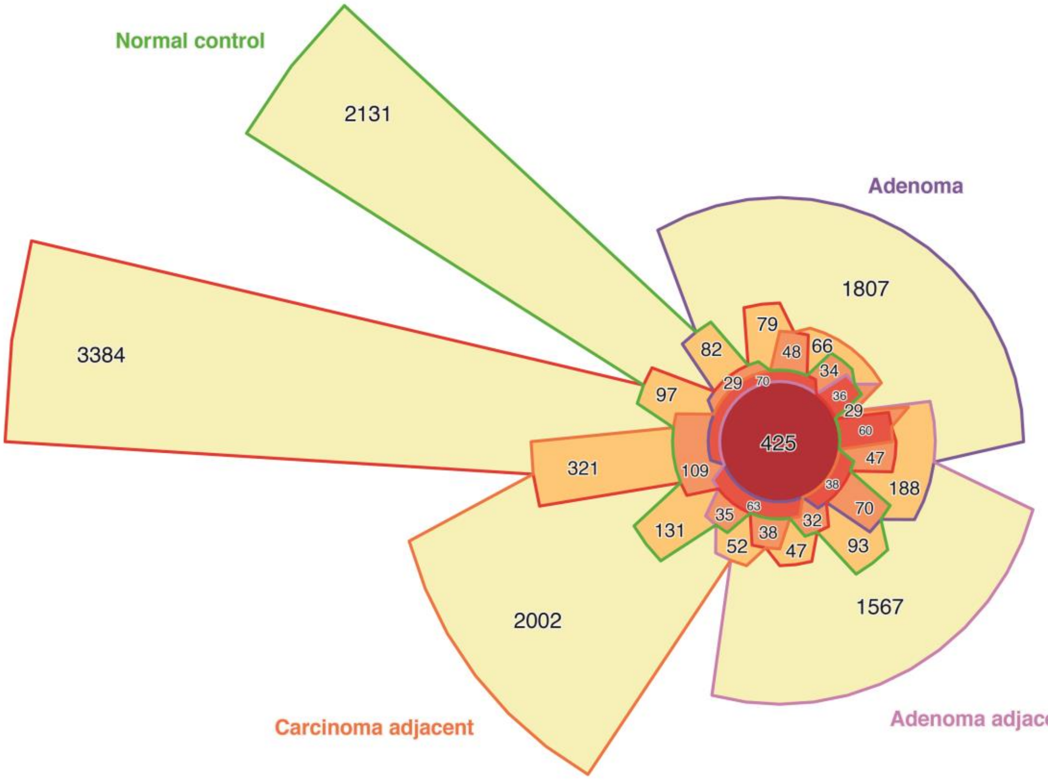

Supplementary Figure 13 | Five-way Venn diagram showing the distribution of 97% OTUs shared among colorectal mucosal phenotypes. Number of shared sequence clusters are provided in the intersected areas.

PATIENT CHARACTERISTICS	NORMAL COLON	COLORECTAL ADENOMA	COLORECTAL CARCINOMA	Adjusted *p*-value (Bonferroni)
**Age, years (mean ± s.d.)**	60.13 ± 5.99	67.32 ± 8.80	67.85 ± 13.18	< 0.001
**Gender (*n*, percent)**				1
Male	25 (40.98)	21 (44.68)	31 (59.62)	
Female	36 (59.02)	26 (55.32)	21 (40.38)	
**BMI, kg/m^2 (mean ± s.d.)**	22.99 ± 2.75	24.02 ± 3.95	22.60 ± 2.79	1
**Chronic alcohol use (*n*, percent)**	3 (4.92)	5 (10.64)	10 (19.23)	1
**Smoking history (*n*, percent)**				1
Chronic smoker	2 (3.28)	4 (8.51)	12 (23.08)	
Current non-smoker	0 (0.00)	8 (17.02)	14 (26.92)	
**First degree family history of CRC (*n*, percent)**	2 (3.28)	6 (12.77)	1 (1.92)	1
**Diabetes mellitus (*n*, percent)**	2 (3.28)	2 (4.26)	4 (7.69)	1
**Anatomic origin (*n*, percent)**				
Proximal	27 (44.26)	20 (42.55)	13 (25.00)	1
Cecum	2 (3.28)	3 (6.38)	2 (3.85)	1
Ascending colon	25 (40.98)	11 (23.40)	10 (19.23)	1
Hepatic flexure	0 (0.00)	2 (4.26)	1 (1.92)	1
Transverse colon	0 (0.00)	6 (12.77)	1 (1.92)	0.121
Distal	34 (55.74)	27 (57.45)	39 (75.00)	1
Splenic flexure	0 (0.00)	1 (2.13)	0 (0.00)	1
Descending colon	10 (16.39)	7 (14.89)	3 (5.77)	1
Sigmoid colon	2 (3.28)	7 (14.89)	10 (19.23)	0.757
Rectosigmoid junction	0 (0.00)	2 (4.26)	6 (11.54)	0.279
Rectum	22 (36.07)	8 (17.02)	19 (36.54)	1
**TNM staging, AJCC 7th Edition (*n*, percent)**				

Zero	–	–	1 (1.92)	–
I	–	–	11 (21.15)	–
II	–	–	14 (26.92)	–
III	–	–	14 (26.92)	–
IV	–	–	12 (23.08)	–
**Histomorphologic type (*n*, percent)**				
Sessile-serrated	–	3 (6.38)	–	–
Tubular	–	28 (59.57)	–	–
Tubulovillous	–	15 (31.91)	–	–
Villous	–	1 (2.13)	–	–
**Grade of dysplasia (*n*, percent)**				
Low	–	35 (74.47)	–	–
High	–	12 (25.53)	–	–

Supplementary Table 1 | Overview of 16S rRNA discovery cohort. Multiple group comparisons for categorical and continuous variables were performed by Chi-squared/Fisher's exact tests and Kruskal-Wallis rank-sum tests, respectively.

PATIENT CHARACTERISTICS	NORMAL COLON	COLORECTAL ADENOMA	COLORECTAL CARCINOMA	Adjusted *p*-value (Bonferroni)
**Age, years (mean ± s.d.)**	41.28 ± 7.87	55.80 ± 11.36	61.34 ± 9.97	1
**Gender (*n*, percent)**				0.086
Male	10 (40.00)	32 (78.05)	26 (52.00)	
Female	15 (60.00)	9 (21.95)	24 (48.00)	
**Anatomic origin (*n*, percent)**				
Proximal	0 (0.00)	11 (26.83)	24 (48.00)	0.031
Distal	25 (100.00)	30 (73.17)	36 (72.00)	1
**TNM staging, AJCC 7th Edition (*n*, percent)**				
Zero	–	–	0 (0.00)	–
I	–	–	6 (12.00)	–
II	–	–	17 (34.00)	–
III	–	–	25 (50.00)	–
IV	–	–	2 (4.00)	–
**Histomorphologic type (*n*, percent)**				
Sessile-serrated	–	2 (4.88)	–	–
Tubular	–	27 (65.85)	–	–
Tubulovillous	–	11 (26.83)	–	–
Villous	–	0 (0.00)	–	–
**Grade of dysplasia (*n*, percent)**				
Low	–	14 (28.00)	–	–
High	–	6 (12.00)	–	–
NA	–	21 (42.00)	–	–

Supplementary Table 2 | Overview of real-time PCR validation cohort. Multiple group comparisons for categorical and continuous variables were performed by Chi-squared/Fisher's exact tests and Kruskal-Wallis rank-sum tests, respectively.
